# Bone Healing by Using Ilizarov External Fixation Combined with Flexible Intramedullary Nailing versus Ilizarov External Fixation Alone in the Repair of Tibial Shaft Fractures: Experimental Study

**DOI:** 10.1155/2014/239791

**Published:** 2014-10-14

**Authors:** A. V. Popkov, N. A. Kononovich, E. N. Gorbach, S. I. Tverdokhlebov, Y. M. Irianov, D. A. Popkov

**Affiliations:** ^1^Laboratory for Limb Lengthening and Deformity Correction, Russian Ilizarov Scientific Centre for Restorative Traumatology and Orthopaedics, 6 M. Ulianova Street, Kurgan 640014, Russia; ^2^Laboratory of Morphology, Russian Ilizarov Scientific Center for Restorative Traumatology and Orthopedics, 6 M. Ulianova Street, Kurgan 640014, Russia; ^3^National Research Tomsk Polytechnic University, 30 Lenin Avenue, Tomsk, Russia

## Abstract

*Purpose*. Our research was aimed at studying the radiographic and histological outcomes of using flexible intramedullary nailing (FIN) combined with Ilizarov external fixation (IEF) versus Ilizarov external fixation alone on a canine model of an open tibial shaft fracture. *Materials and Methods*. Transverse diaphyseal tibial fractures were modelled in twenty dogs. Fractures in the dogs of group 1 (*n* = 10) were stabilized with the Ilizarov apparatus while it was combined with FIN in group 2 (*n* = 10). *Results*. On day 14, a bone tissue envelope started developing round the FIN wires. Histologically, we revealed only endosteal bone union in group 1 while in group 2 the radiographs revealed complete bone union on day 28. At the same time-point, the areas of cancellous and mature lamellar bone tissues were observed in the intermediary area in group 2. The periosteal layers were formed of the trabeculae net of lamellar structure and united the bone fragments. The frame was removed at 30 days after the fracture in group 2 and after 45 days in group 1 according to bone regeneration. *Conclusion*. The combination of the Ilizarov apparatus and FIN accelerates bone repair and augments stabilization of tibial shaft fractures as compared with the use of the Ilizarov fixation alone.

## 1. Introduction

The rise in the number of severe injuries due to high energy and traffic trauma has resulted in the search for more efficient and faster methods of fracture and nonunion repair. External and internal fixation methods have been widely used for their operative management and evolved a lot [1]. Recently, the techniques that combine external fixation and intramedullary nailing have proven to be more efficient in regard to outcome, patients' comfort, and a shorter inpatient stay both for cases of fractures and for orthopaedic diseases [2–5]. Most studies of their application demonstrate the radiographic and clinical findings that confirm the reasonability of their combination. However, there is little fundamental experimental research that could reveal the consolidation process by the application of the combined techniques for fracture healing.

Our research was aimed at revealing the differences in radiographic and histological outcomes of bone repair by using flexible intramedullary nailing (FIN) combined with the Ilizarov external fixation (IEF) versus the Ilizarov external fixation alone on a canine model of an open diaphyseal tibial fracture.

## 2. Materials and Methods

Open transverse diaphyseal tibial fractures (Gustilo type I) [6] were modelled in twenty adult mongrel dogs aged from one to 5 years that had their tibial physes closed. Their mean body mass was 20 ± 2.9 kg.

The tibia was broken from a soft tissue incision on the medial side at the level of the middle diaphysis. The osteotomies of the tibia and fibula were performed with a chisel. Tibial transverse fractures were obtained in all cases.

**Figure 1:**
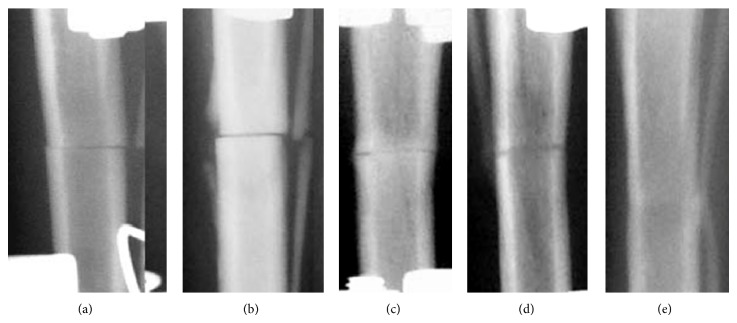
Group 1 radiographs: (a) day 7, (b) day 14, (c) day 30, (d) day 45, and (e) day 75.

**Figure 2:**
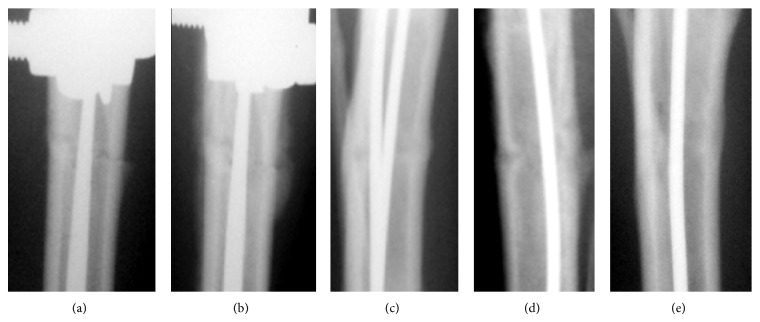
Group 2 radiographs: (a) day 7, (b) day 14, (c) day 30, (d) day 45, and (e) day 75.

Fractures in group 1 (*n* = 10) were stabilized with the Ilizarov apparatus (Figure 1) while in group 2 dogs (*n* = 10) the IEF was combined with FIN (Figure 2). Soft tissues were stitched.

We used two 1.8 mm hydroxyapatite-coated titanium wires for performing tibial FIN. The wire diameter measures from 20 to 25% of the bone marrow cavity diameter. Such a range of the wire diameter is used for bone lengthening with the combination of IEF and FIN [4]. They were inserted from the medial and lateral sides at the level of the tibial tuberosity and were introduced into the medullary cavity by pushing them down to the level of the distal metaphysis. The wires were cut at the entrance into the bone, and the wound was closed. Those wires remained in the canals of group 2 dogs during the entire experiment.

The Ilizarov apparatus assembly comprised four external rings. Two crossed Kirschner wires were inserted at each ring level and attached to the rings. The rings were connected with threaded rods. This variant of IEF assembly is considered to be stable enough both for experimental studies and for clinical use [4, 7–9]. Full weight bearing was allowed immediately for all dogs without any holder.

The postoperative radiographic images showed transverse fractures in the middle third of the tibial diaphyses (Figures 1(a) and 2(a)). The radiographic images and histological samples were studied on days 14, 28, 45, and 75 after the intervention. The Ilizarov fixator was dismounted after 30 days in group 2 and after 45 days in group 1 according to the quality of regeneration.

Radiographic views were taken with the aid of VEP X Technology Premium Vet device (Spain) on the day of the operation and day 7. Further on, they were taken by the end of each time-point before the histological material was harvested. Two dogs of each group were euthanized after 14, 28, 45, and 75 postoperative days using lethal doses of sodium thiopental for preparing histological sections. Two dogs in each of the groups were not euthanized.

The bone tissue samples for histological examination were sections sawed longitudinally along the tibial axis. They were placed into the mixture of aldehyde fixators and picric acid and dehydrated by ethyl in ascending concentrations. One section part was decalcified in the mixture of formic and hydrochloric acid. The second section part was not decalcified but was immersed into araldite. Histotopographic sections were stained with hematoxylin eosin and according to Van Gieson stain. The surfaces of araldite blocks were polished, sputtered with the platinum and palladium alloy in the ionic vacuum sputtering system IB-6 (Eiko, Japan), and were studied with the aid of the electron probe microanalyzer INCA-200 Energy (Oxford Instruments, UK).

Interventions, animal care, and euthanasia conformed to the requirements of the European Convention for the Protection of Vertebrate Animals used for Experimental and other Scientific Purposes (Strasbourg, 18.03.1986), principles of laboratory animal care (NIH publication number 85-23, revised 1985), and the national laws. The study was approved by the ethics board of the institution.

## 3. Results

### 3.1. Radiographic Findings

The periosteal response and shadows of endosteal bone formation at the fracture site were seen after two weeks in group 1 (Figure 1(b)) and after one week in group 2 (Figure 2(a)). The fracture lines were still well seen.

After 14 days, the fragments' ends in group 2 seemed vague and the osteotomy line was not well distinguished. Shadows of endosteal response were expressed and the periosteal response at the level of the fracture was presented by shadows of uneven and unclear contours which were 2.8 ± 0.2 mm thick and 14.7 ± 1.3 mm long. In most cases, the periosteal layers bridged the osteotomy line (Figure 2(b)).

In group 1, homogenous shadows were seen in the fracture gaps by day 28 (Figure 1(c)). Those shadows of endosteal regeneration were 3.5 mm deep into both fragments while the compact periosteal layer was 1.2 mm high. Complete bone union with the periosteal layer that was completely reduced was observed in this group by day 45 (Figure 1(d)). On postoperative day 75, the axis was aligned. The fracture line was hardly seen and both endosteal and periosteal reactions at the osteotomy level were not noted (Figure 1(e)).

In group 2, the radiographs revealed bone union on day 28 (Figure 2(c)). The fragments' ends merged with the fracture line so that it was hardly seen. Compact periosteal layers united the proximal and distal fragments. Endosteal response was seen along the FIN wires. The Ilizarov fixator was taken off in the remaining dogs of this group on day 30. On day 45 the osteotomy line featured only single light areas. Endosteal response was observed along the FIN wires but the periosteal layers decreased (Figure 2(d)). On day 75, we observed the unified medullary cavity and continuous cortex along the bone circumference at the fracture level (Figure 2(e)). The osteotomy line and periosteal reaction were not visualized. However, the shadows in the bone marrow cavity showed more contrast radiographically. Bone fragments in both groups were stable during the entire experiment period. Intramedullary wires were not displaced. Infection was not observed.

### 3.2. Histological Findings

#### 3.2.1. Group 1

**Figure 3:**
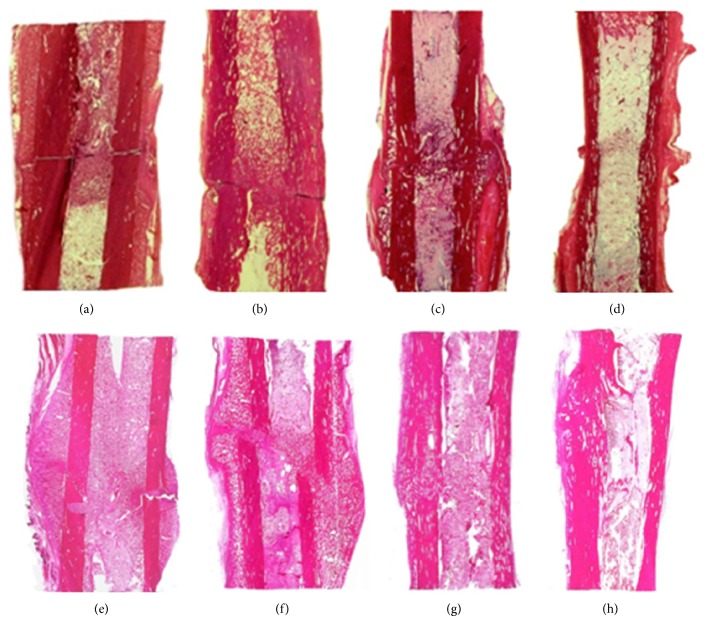
Tibial histotopograms. Group 1 (upper row) and group 2 (lower row): (a, e) day 14; (b, f) day 28; (c, g) day 45; and (d, h) day 75. Hematoxylin eosin staining, ×1.5.

**Figure 4:**
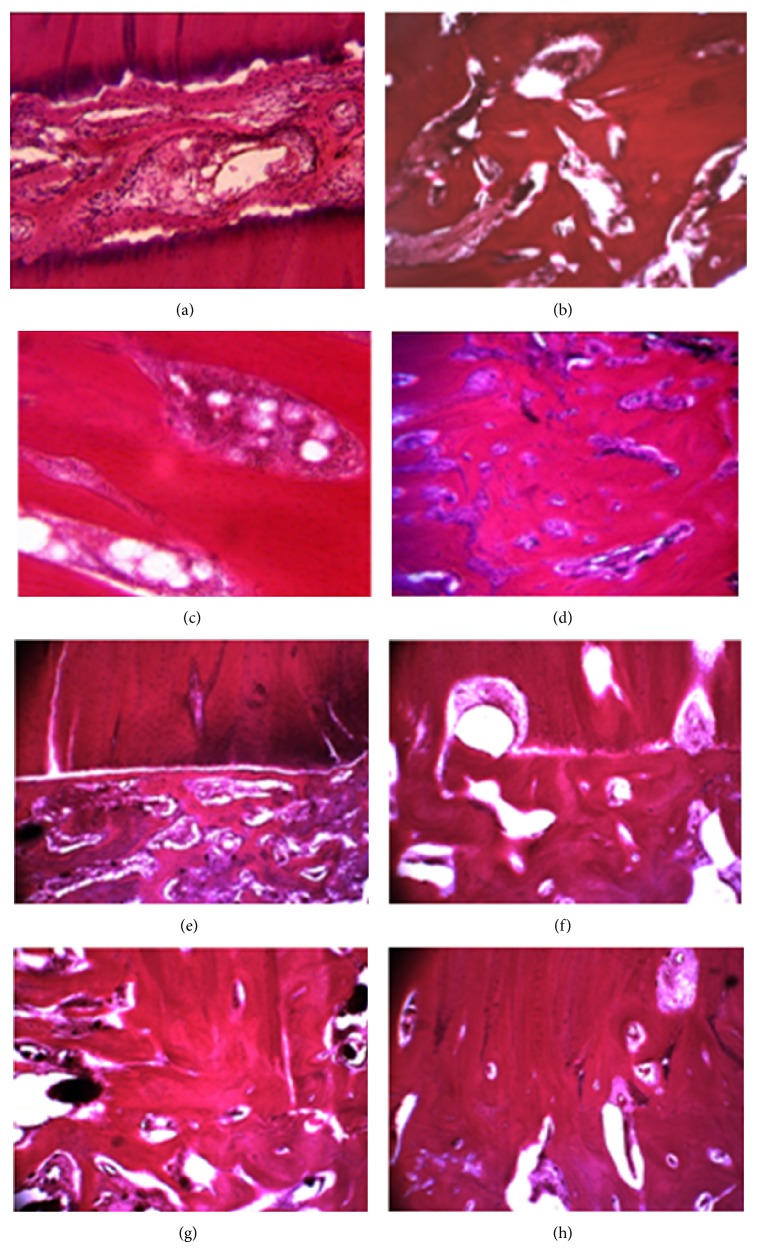
Microphotos of the intermediary regeneration areas. Group 1 (a–d) and group 2 (e–h): (a, e) day 14; (b, f) day 28; (c, g) day 45; and (d, h) day 75. Hematoxylin eosin staining. Magnification: (a, b, d–h) ×40 and (c) ×63.

The histological study on day 14 revealed loose fibrous connective tissue and weakly mineralized bone trabeculae of reticulofibrous structure in the intermediary area of the fracture site (Figure 3(a)). The trabeculae were oriented in the perpendicular direction relative to the long bone axis (Figure 4(a)). Periosteal regeneration was presented by cancellous bone tissue of the middle-sized cellulae and areas of nonmineralized connective tissue. The distal and proximal periosteal layers were not bridged. The endosteal regeneration was also of spongy structure but the cellulae were of the middle or large sizes with insertions of chondroid areas and fibrous cartilage.

On day 28, we revealed endosteal bone union (Figure 3(b)). There were massive reticulofibrous trabeculae of cancellous bone in the gap between the fragments and loose fibrous connective tissue in the space between the trabeculae (Figure 4(b)). Periosteal cancellous bone was undergoing reorganization into the compact structure and its proximal and distal portions started to unite.

Complete bone union was seen on day 45 (Figure 3(c)) when the periosteal, intermediary, and endosteal regeneration areas featured reticulofibrous cancellous bone with red bone marrow in the space between the trabeculae (Figure 4(c)).

On day 75, the tibia consolidated through lamellar bone tissue in the intermediary area between the fragments' ends (Figure 3(d)). The new cortex at the fracture level was formed of osteons that were not oriented regularly yet (Figure 4(d)). The medullary cavity was filled with hematopoietic and fatty bone marrow.

#### 3.2.2. Group 2

**Figure 5:**
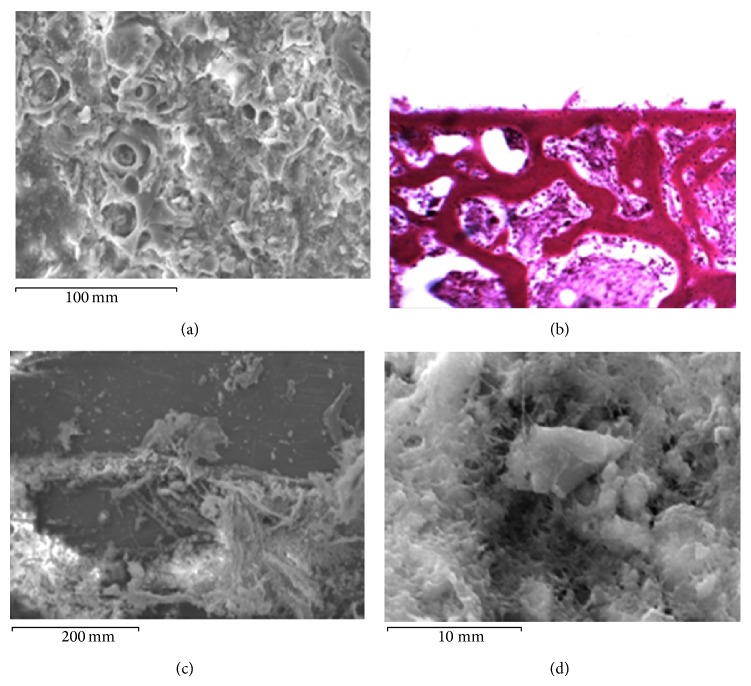
Structural features of the FIN wire surface and its adjacent area: (a) architectonics of the FIN wire, SEM ×1300; (b) osseoosteoid trabecular net envelope in the area adjacent to FIN wire, day 14, hematoxylin eosin staining, ×100; (c) fixation of collagen fibres of the osseoosteoid envelope to the rough FIN wire surface, day 14, SEM ×400; and (d) adhesion of osteogenic cells to the FIN wire surface and amorphous bone matrix in the pericellular space, SEM ×2500.

The sections on day 14 showed a similar picture as in group 1 (Figure 3(e)). However, the portion of bone tissue in the intermediary area was bigger as compared to group 1 (Figure 4(e)). The difference between the groups was the envelope formed of bone tissue that started developing round the FIN wires and this process persisted till the end of the experiment (Figure 2(a)). That envelope was made of a trabecular net that represented reticulofibrous bone tissue and osteoids (Figure 5(a)). In the spaces between the trabeculae of the envelope there were areas of granulated tissue that had numerous vessels and perivasculocytes that featured different stages of differentiation. The collagen fibres that formed the fibrous frame of the osseoosteoid envelope were attached to the rough HP-coated wire surface (Figure 5(b)) and were connected between each other with tiny spreading fibres that built the fibrous frame (Figure 5(c)).

On day 28, cancellous and mature lamellar bone tissues were observed in the intermediary area (Figures 3(f) and 4(f)). Periosteal layers that were formed of the trabeculae net of lamellar structure united the bone fragments. The rough fibrous bone tissue in the envelope round the wires kept developing into a more mature and mineralized lamellar bone. Adhesion of osteogenic cells and amorphous bone matrix were also seen (Figure 5(d)).

Complete periosteal, intermediary, and endosteal bone union was observed on day 45 (Figure 3(g)). In the intermediary area, the bone fragments were united through the trabecular net of narrow cellulae and osteons that had various maturity stages (Figure 4(g)). The cortical layer was undergoing the phase of compactization. The bony envelope round the FIN wires was presented by the compact bone of lamellar structure in which osteons were under formation as well as by cancellous bone tissue that was filling in the medullary cavity of bone fragments and fixed it like a rod.

**Figure 6:**
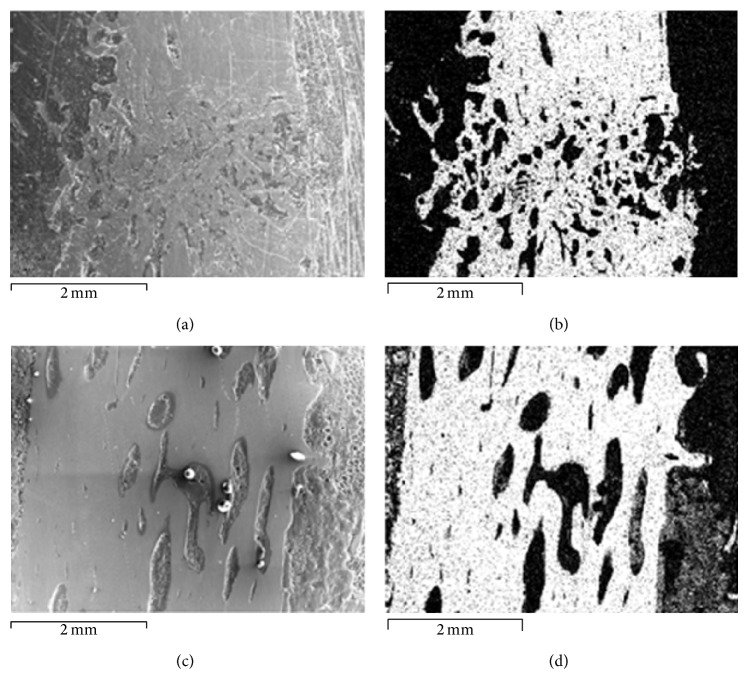
Bone regeneration in the intermediary area on day 75. Group 1 (upper row) and group 2 (lower row): (a, c) scans, ×20; (b, d) calcium distribution in the intermediary area by electron probe X-ray microanalysis.

The histotopograms on day 75 showed the regenerated bone that had a more typical and organic structure of the regular bone tissue than in group 1. It was expressed by a more compact structure of the new bone on the fracture site (Figures 3(h) and 4(h)). Electron probe X-ray microanalysis on that day revealed a better calcium saturation at the fracture level than in group 1 (Figure 6).

## 4. Discussion

Open fractures of the tibial shaft often occur after a high energy direct trauma and their operative treatment is demanding [7, 10]. The studies that compare different methods of their fixation show high rate of nonunion when managed by the available methods of stabilization such as intramedullary nailing, plating, or external fixation when they are used independently [1, 2].

The Ilizarov techniques have shown to be useful in the management of difficult fractures and nonunions of the tibia but practicing surgeons call attention to their main drawbacks such as the long time the patients have to spend with the fixator on, much discomfort, and pin tract infections [3, 4]. Therefore, the techniques that combine the external device with intramedullary nailing have been advocated to avoid these problems. The combined techniques resulted in the reduction of the usual IEF duration and good union rates [3–5]. There were many clinical studies of using the external fixator over the nail or FIN for orthopaedic conditions and injuries [4, 5, 11, 12].

In fact, the IEF provides optimal reduction of complex tibial fractures while internal nailing augments their stabilization. Accurate reduction and stabilization will both be gained by such a combination. When primary consolidation happens, the external fixator can be removed and the nail can be left inside. However, there is much discussion of the condition of vascularisation during reaming and nailing due to possible damage of the medullary vessels and content. Undoubtedly, nailing affects the osteogenic potential of the bone marrow. Unreamed nailing is favoured nowadays as it produces less compression of the medullary contents though other studies show higher consolidation rates with reamed nailing [2, 13].

Bone regeneration has been widely studied using a canine model [9, 14, 15]. Our study has combined the Ilizarov fixator assembly for diaphyseal fractures with FIN that uses a couple of thin wires aimed at a lesser damage of the medullary content as compared to a single nail. The optimal diameter of such wires was calculated [16] and their combination with the Ilizarov fixator was already used and showed good outcomes in limb lengthening situations both experimentally and clinically [4, 9].

The available literature does not give a clear picture of the process of bone healing during the repair of shaft fractures and management of nonunions when combined external and intramedullary fixation is used to explain how the healing process develops.

Our study demonstrated that the healing process of a diaphyseal tibial fracture was faster and was completed by day 28 in group 2 whereas in group 1 it continued till day 45. The time difference of 15 days in the confirmed bone union in our groups is obviously in favour of the combined technique. The quality of regeneration was better in group 2 at all time-points studied. The endosteal bone formation along the wires was an additional reinforcing factor. Moreover, we suppose that the endosteal envelope was also an additional inducer of the periosteal osteogenesis and fragments' ends cambial cells osteogenesis.

We assume that the possible mechanism of the effect of using FIN is also associated with a prolonged formation of granulation tissue foci in the marrow cavity that stimulate the population of osteoproductive cells and angiogenesis that result in the activation of bone reparation.

Another advantage of the use of FIN technology is the fact that fracture consolidation in group 2 of our series was of primary type that featured neither cartilaginous nor connective tissues. No reaming is required in our technology. No damage to the intraosseous artery with the wires was observed. Moreover, the procedure of introducing intramedullary wires and taking them out is not technically demanding and does not injure the tissues that could affect the knee joint. Therefore, no knee problems may be expected. Those problems were not encountered by using the FIN technology for limb lengthening [4, 9].

## 5. Conclusion

We conclude that our experimental study proves that the combination of the Ilizarov apparatus and FIN augments fixation stability of bone fragments, accelerates the repair of tibial shaft fractures, and can be used in clinical settings. This combined technique does not contradict the biological principles of the Ilizarov method.
